# Feature binding and detachment in psychosis: A virtual reality study

**DOI:** 10.1016/j.scog.2025.100376

**Published:** 2025-06-25

**Authors:** A.J. (Ante) Schlesselmann, G.H.M. (Marieke) Pijnenborg, S.A. (Saskia) Nijman, W. (Wim) Veling, R.J.C. (Rafaele) Huntjens

**Affiliations:** a Department of Clinical Psychology and Experimental Psychopathology, University of Groningen, Groningen, the Netherlands; b Department of Clinical and Developmental Neuropsychology, University of Groningen, Groningen, the Netherlands; c Department of Psychotic Disorders, GGZ Drenthe, Assen, the Netherlands; d Early Detection and Intervention Team Haaglanden, PsyQ, Parnassia Psychiatric Institute, The Hague, the Netherlands; e Department of Psychiatry, UMCG, University of Groningen, Groningen, the Netherlands

**Keywords:** Detachment, Dissociation, Psychosis, Schizophrenia

## Abstract

**Background:**

Schizophrenia spectrum disorders (SSDs) significantly impact daily functioning, particularly through cognitive deficits like memory impairment. Traditionally attributed to neurobiological factors, recent evidence highlights the role of psychological processes like detachment, which may disrupt episodic memory encoding and retrieval by impairing feature binding. This study used a virtual reality (VR) paradigm to explore whether state detachment in SSDs is linked to impaired feature binding for adverse stimuli.

**Methods:**

Twenty-five SSD patients from Dutch mental health centers and 25 individuals from the general population participated. Using an immersive VR paradigm, participants navigated a virtual shopping mall, interacted with 3D avatars, and identified their emotional facial expressions. Three memory tests followed: avatar identity recognition (basic memory), binding emotional expressions to avatars, and binding avatar identity to the encounter's temporal order. State detachment was measured using the Clinician-Administered Dissociative States Scale (CADSS).

**Results:**

The SSD and comparison group did not display significant performance differences in any of the three feature binding tasks. However, across groups, results indicated that higher state detachment levels corresponded with worsened identity-emotion binding specifically for angry faces.

**Conclusion:**

The present study provides tentative empirical support for the role of detachment in feature binding deficits for angry faces both in the patient and comparison group. Future studies should further explore the impact of psychological mechanisms like detachment on memory dysfunction, particularly regarding aversive stimuli.

## Highlights

• Memory deficits affect daily functioning in SSDs.
• Detachment may impact feature binding for episodic memory.
• Participants identified emotions of 3D avatars in a VR shopping mall.
• Memory tests assessed avatar identity and emotion binding.
• Higher detachment linked to poorer binding for angry faces.

## 1 Introduction

Schizophrenia spectrum disorders (SSD) are multifaceted mental disorders marked by a disruption in thought, perception, and behaviour (American Psychiatric Association, 2022). Among the multitude of symptoms associated with SSD, cognitive impairments, particularly in memory, play a critical yet often underappreciated role in the daily challenges faced by individuals with these disorders (Cervellione et al., 2007; Green, 1996; Shamsi et al., 2011). Meta-analyses revealed impairments to be common and widespread for short-term memory as well as long-term memory (Aleman et al., 1999; Gebreegziabhere et al., 2022). Deficits in episodic memory are particularly pronounced in SSD with moderate to large effect sizes found across different studies with a variety of research designs (Berna et al., 2015). Notably, memory impairments are among the strongest predictors of decreased social functioning and can significantly impact quality of life, making them an important factor to consider in interventions for SSDs (Green, 1996; Bowie and Harvey, 2006). Hence, it is paramount to investigate the factors influencing memory deficits experienced by individuals with SSDs. In line with this objective, the present study aims to further our understanding of memory impairment by complementing it through the consideration of a potential psychological factor impacting episodic memory performance.

A psychological process that has been shown to be associated with memory functioning is dissociation (Ehlers and Clark, 2000; Holmes et al., 2005). Dissociation is defined as “a lack of normal integration of thoughts, emotions, and experiences into consciousness and memory” (American Psychiatric Association, 2022). While dissociation is a defining characteristic of dissociative disorders, it encompasses a range of transdiagnostic experiences appearing in various disorders and as highlighted by recent reviews, also in the SSD population (Lyssenko et al., 2018; Renard et al., 2017).

Interestingly, the phenomenological experiences characterizing dissociative symptoms have recently been described as a core phenotypic marker of SSD. These experiences, referred to in the literature as basic-self disturbance, highlight their essential role in the phenomenology of SSD (Feyaerts and Sass, 2024; Nelson and Sass, 2017). Expanding on the work of Allen and colleagues (Allen et al., 1999), Holmes and colleagues (Holmes et al., 2005) describe how dissociative experiences, particularly a set of symptoms subsumed under the term *detachment*, may exert an influence on the encoding process of episodic memory. Under normal circumstances, sensory perceptions experienced during an event are integrated collectively through a constructive process (Baddeley et al., 2014). This integration results in a coherent memory of the episode. However, individuals experiencing detachment often report sensations akin to being spaced-out or in a dream-like altered state of consciousness, marked by a sense of disconnection from elements of everyday reality. This may include a separation from one's sense of self (depersonalization) but also from the external world (derealization) often in response to adverse events (Holmes et al., 2005). While initial heightened arousal typically facilitates the encoding of emotional material (Kensinger, 2009). In contrast, dissociative symptoms as a means of avoiding or disengaging from adverse stimuli may obstruct a coherent encoding process of information.

A crucial process for the formation of coherent memories is *feature binding*. The term refers to the capacity to combine various sensory characteristics of an event or object (Treisman, 1996, Treisman, 1998). Two types of feature binding are temporal and contextual binding (Treisman and Gelade, 1980). Temporal binding involves linking the chronological sequence of events and is required, for instance, when attempting to accurately remember the order of elements of a previous encounter. Contextual binding refers to connecting events or objects with their surrounding circumstances, which aids in event recall and interpretation. The binding of information may be compromised in individuals experiencing symptoms of detachment. This might particularly apply for adverse stimuli with the innate potential to elicit experiences of detachment. For instance, an individual experiencing detachment during an adverse event, such as a mugging incident, might be unable to later identify the assailant in a police lineup or correctly recall the order of events, as features of the perpetrator such as clothing, facial details, their voice, etc., were not encoded into one coherent episode of memory.

While not previously articulated as examples of feature binding, earlier research yielding mixed outcomes is important to consider when examining the link between detachment and feature binding. In one study in a non-clinical population, participants viewed an aversive film and were then tasked with arranging clips from the movie into their original sequence (Kindt and van den Hout, 2003). Neither the original study nor its replication found a significant link between the level of dissociation experienced during the viewing and the performance in temporal ordering (Kindt and van den Hout, 2003; Kindt et al., 2005). However, in a similar experiment, Giesbrecht and colleagues (Giesbrecht et al., 2010) discovered that individuals diagnosed with depersonalization disorder had more difficulty accurately sequencing the clips compared to a group without symptoms. In a study by Huntjens and colleagues (Huntjens et al., 2015), participants were presented with sequences of pictures varying in emotional arousal and valence. After viewing each sequence, they were tasked with rearranging all the pictures, given to them simultaneously, into their original order. The findings revealed a negative correlation between the level of state anxiety experienced during the task and the number of errors made while sorting highly arousing, negative materials indicating impaired temporal context binding. However, this effect was not observed in relation to state dissociation. Notably, one potential limitation of their study, as suggested by the authors, was that the picture stimuli were intrinsically unrelated, making them somewhat artificial.

## 2 The present study

This study seeks to enhance previous research in two key areas. Firstly, unlike prior studies that utilized a non-specific measure of dissociation, we propose a more targeted approach by focusing specifically on the experience of detachment. This focus follows theoretical assumptions of detachment as the dissociation symptom dimension most closely associated with disintegrated encoding of information following exposure to adverse stimuli (Holmes et al., 2005). This is in line with the results obtained by Giesbrecht and colleagues (Giesbrecht et al., 2010) in the sample of individuals with depersonalization disorder reflecting the epitome of detachment manifestation. Secondly, while previous research effectively employed film clips and emotional picture stimuli to study the impact of dissociation on feature binding performance, these methods only approximate real-world scenarios where effective feature binding in the face of aversive stimuli is most relevant. As one potential improvement on the previous studies, we employed virtual reality (VR) technology as a more ecologically valid paradigm, aligning as closely as possible with daily life experiences of individuals with SSDs.

Proven safe and tolerable for SSD patients, VR has found successful application in the investigation of cognitive processes as well as treatment and assessment (Macedo et al., 2015; Pot-Kolder et al., 2018; Rus-Calafell et al., 2018; Zawadzki et al., 2013). To test how experiences of detachment in the moment may be associated with impaired feature binding, an aversive stimulus capable of eliciting *state detachment* is required. For this purpose, we used angry faces. Not only is an angry expression universally recognized and commonly perceived to signal potential threat (Fox et al., 2000; Izard, 1994; Ohman and Soares, 1993), but it also features high ecological validity as it is encountered in everyday life.

The exposure to adverse stimuli such as angry faces, does not solely provoke state detachment but also may trigger anxiety. Moreover, clinical models of depersonalization disorder as well as empirical evidence highlight the association of anxiety and dissociation (Huntjens et al., 2015; Hunter et al., 2003; Sterlini and Bryant, 2002). Hence, when exploring the potential influence of state detachment on feature binding, the effects of anxiety are an important consideration as well. Consequently, we also included a measure of state anxiety in our assessment of the association of state detachment and feature binding impairment.

We hypothesized a) that individuals with an SSD exhibit deficits in feature binding compared to comparisons from the general population, and b) and that an individual's level of state detachment is negatively associated with feature binding performance, particularly for angry faces, even while controlling for state anxiety.

## 3 Method

### 3.1 Participants

The study was a part of the Dynamic Interactive Social Cognition training in Virtual Reality project (DiSCoVR; see (Nijman et al., 2019; Nijman et al., 2023)) which was approved by the Medical Ethical Committee of the UMCG (METc file number: 2017/573, ABR: NL63206.042.17). A total of 50 Dutch participants were recruited across two studies for the present study. Twenty-five of those were SSD patients from two of the five participating mental health centers. Participants had either received a diagnosis of a psychotic disorder within the past three years or their diagnosis was confirmed using the Mini International Neuropsychiatric Interview Plus 21 (Sheehan et al., 1998). Further DiSCoVR inclusion criteria were an indication of impaired social cognition by the treating therapist and an age between 18 and 65. There was an equal number of comparison subjects from the general population in the same age range, who were recruited through social media. Exclusion criteria for both the clinical and comparison group were insufficient proficiency of the Dutch language, an estimated premorbid IQ below 70 which was assessed via the Dutch version of the National Adult Reading Test (Nelson and Willison, 1991; Schmand et al., 1992) as well as relevant neurological disorders such as dementia, epilepsy or organic brain damage (Nijman et al., 2019). Further, individuals in the comparison group were excluded if they had ever received a diagnosis or treatment for any mental health problems. Participants were matched on age, gender and education level.

### 3.2 Instruments

#### 3.2.1 State trait anxiety inventory

The STAI (Spielberger, 1970) is used to assess state and trait anxiety. For the purpose of our study the state anxiety scale (SAI) of the STAI was used, containing 20 items. One half of the set includes items such as “I am tense” whereas the other half is reverse coded and encompasses items such as “I feel calm”. The statements of the questionnaire are scored on a four-point Likert scale, ranging from ‘absolutely not’ (1) to ‘very much’ (4). This measure was selected due to its good psychometric properties (van der Bij et al., 2003), also in SSD samples (Georgiev et al., 2012; Vancampfort et al., 2011). Internal consistency in the current sample was very high as indicated by a Cronbach's alpha of 0.95.

#### 3.2.2 Clinician administered dissociative states scale

The CADSS (Bremner et al., 1998) was developed to measure alterations in state dissociation in clinical populations. It differentiates between three common subdivisions of dissociative experiences, namely, depersonalization, derealization and amnesia. For the present study the CADSS was adapted to the VR experience excluding the eight observer items. The 19 remaining self-report items (e.g. “Do things seem to be moving in slow motion?”) were rated on a five-point Likert scale, ranging from ‘absolutely not’ (0) to ‘extremely’ (4). Of these 19 items, 17 items referred to depersonalization and derealization (i.e., detachment), hence a sum score from these items was used for further analyses and the two items reflecting amnesia (item 14 and 15) were discarded. The 17 selected CADSS self-report items had satisfactory reliability and construct validity (Condon and Lynn, 2014) were previously applied in SSD samples (Bremner et al., 1998; Ahn et al., 2011) and showed moderate internal consistency in the current sample with a Cronbach's alpha of (0.76) for the selected state detachment subscale.

#### 3.2.3 Positive and negative syndrome scale

The PANSS (Kay et al., 1987) is a semi-structured interview consisting of 30 items used to assess symptoms of SSD. Interviewers assign ratings to each item on a 7-point scale, from “absent” (1) to “extreme” (7). The PANSS has demonstrated good validity and reliability (Peralta and Cuesta, 1994). In this sample, scores of the negative and positive symptom subscales (7 items each) were utilized to determine symptom severity. Both the positive and negative subscale demonstrated acceptable internal consistency with a Cronbach's alpha of 0.55 and 0.62 respectively. This measure was only administered in the SSD group.

### 3.3 Procedure

#### 3.3.1 VR encoding task

**Fig. 1:**
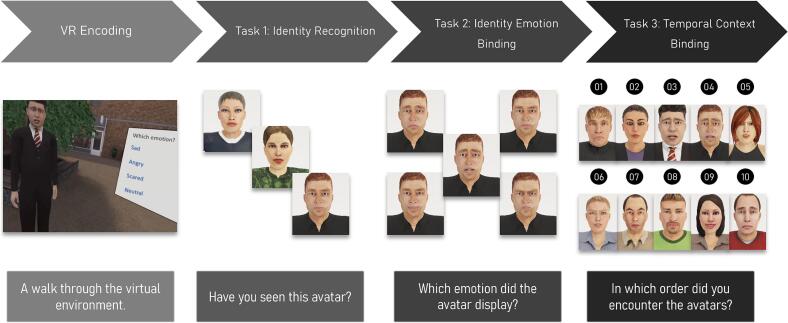
Overview of the experimental phases. The figure illustrates four key stages of the experiment: (a) the encoding phase, where participants navigated the virtual environment, and three memory tasks: (b) recognition of avatar identity, (c) binding emotional expressions to avatars, and (d) binding avatar identity to the temporal order of encounters. These stages depict the core components of the VR paradigm used to assess memory performance.

The encoding task consisted of exploration of a virtual shopping mall, during which participants encountered ten avatars. The participants used an Oculus Rift (Consumer Version 1) VR head-mounted display to experience the shopping mall and moved around the environment by using an Xbox controller. Participants could move forward and backward by using the joystick and turn left or right by turning their head in that particular direction. As can be seen in Fig. 1, upon approach, an avatar would present one of five emotional expressions to a participant (happy, sad, angry, fearful, or neutral). There were two avatars per emotion included in the task. The dialogue box showed four of the five possible emotions in text form and individuals had to select which of the emotions was displayed by the avatar. The participant was given a maximum of two attempts for providing the correct answer. In case the participant provided an incorrect answer twice, the avatar walked away, and the participant could move on to the next one. The avatar also walked away when the participant provided a correct answer. Regardless of participants' answers, the session ended automatically after 10 avatars. Participants in the clinical group had completed a single practice session (<10 min) with the VR environment as part of the DiSCoVR program, whereas the comparison group had not used it before. To ensure that participants from the comparison group could familiarise themselves with the VR headset and controls, a test run featuring only two avatars from a distinc*t-*test set of avatars with neutral expressions was conducted before the main trial session.

After completing the VR task, participants completed the CADSS and SAI in a counterbalanced order. The questionnaire period was set to 10 min to standardize the interval before the recognition task. Participants then completed the three memory tasks. They were not informed that their memory for the avatars or their emotional expressions would later be tested. This incidental encoding design was chosen to preserve ecological validity and to simulate naturalistic social interactions. Lastly, the study protocol accounted for potential discomfort or motion sickness during the VR task, participants could sit down and continue if needed, though no notable cases were observed.

#### 3.3.2 Task 1 Identity recognition

The first assessment represented a basic recognition memory control task where participants had to identify the ten avatars they had seen out of a larger set of 24 avatars. Participants were shown all pictures sequentially with neutral facial expression, one at a time in random order. With each picture shown, participants were asked to indicate whether they saw the avatar presented in the picture during the VR task (i.e., yes/no recognition). To compare group performance, the general hit rate across emotions as well as for angry faces alone was calculated as were measures of sensitivity (d') and decision bias (c) in accordance with signal detection theory for the measure across emotions (Stanislaw and Todorov, 1999).

#### 3.3.3 Task 2 Identity emotion binding

For the second task, which captured identity binding of facial emotional expression and identity of an avatar, participants were presented with five pictures for every one of the ten avatars they had previously encountered in the VR environment. Pictures were shown independent of whether participants correctly identified them in the first recognition task or not. Images featured all five possible emotional expressions (angry, fearful, sad, happy and neutral). Both the avatars and the emotions (per avatar) were presented in a randomized order. The participants were then asked to indicate which of the five pictures matched the expression that a particular avatar presented during the VR task. We calculated a relative hit rate to capture performance across all emotions as well as separately for angry faces. The relative hit rate was calculated by dividing the number of hits by the number of valid trials (i.e., avatars with correctly identified emotion in the encoding phase after max 2 attempts).

#### 3.3.4 Task 3 Temporal context binding

During the final task, temporal feature binding was tested. Participants were presented with the ten avatars they had seen in the VR task in random order, displaying the same facial emotional expressions as in the VR task. Participants were then asked to recreate the order in which they had approached the avatars during their virtual walk. The outcome variable was the amount of correct adjacent pairs of avatars (e.g. avatars 3, 4 or 8, 9 are placed together), thus complete data was required and no specific analysis for angry faces alone could be performed. For an overview of the experimental procedure and task see Fig. 1.

### 3.4 Analysis

All analyses were conducted using SPSS (version 29). Group comparisons between the SSD and comparison group were performed using one-sided independent samples *t*-tests, reflecting our directional hypothesis that the SSD group would show reduced memory performance. These comparisons were conducted for each of the three memory tasks (identity recognition, identity-emotion binding, and temporal context binding), both across all emotional expressions and specifically for angry faces where applicable. Statistical assumptions were assessed prior to analysis. In cases where unequal variances were detected, Welch's *t*-test was used.

To test the association between state detachment and feature binding performance (identity-emotion binding task), four multiple regression models were estimated. Two models predicted overall identity-emotion binding performance (with and without controlling for recognition performance), and two models focused specifically on trials involving angry faces (again with and without recognition performance as a covariate). All regression models included age, gender, education level, and state anxiety as covariates. Assumptions were assessed prior to analysis.

For identity recognition performance, sensitivity (d′) and decision bias (c) were calculated in accordance with signal detection theory. Continuity corrections were applied when hit or false alarm rates were equal to 0 or 1.

## 4 Results

One participant from the patient group had to be excluded due to an error in the VR software and a participant from the comparison group had to be excluded due to an experimenter error in the second task. Another participant had to be excluded as they indicated a history of mental disorder. Assumptions for linear regression were assessed, and no evidence for a violation of assumptions was detected. All analyses were run in SPSS (v29).

See Table 1 for an overview of demographic variables. Neither age, gender nor education level were significantly different between the groups. The SSD group had a significantly higher score on state detachment than general population comparisons (*t*(45) = 2.61, *p* = .012), with the SSD group scoring 14 (*SD* = 6.9) and the comparison group 9.1 (*SD* = 5.8) on average. Similarly, the SSD group displayed significantly higher state anxiety (*t*(35.6) = 5.21, *p* < .001) with 40 (*SD* = 10.7) on average for the SSD group and 27.1(*SD* = 5.7) for the comparison group. Due to unequal variances, Welch's *t*-test for independent groups was applied for the state anxiety measure.

**Table 1:** Demographics.

	SSD	Comparison	Test	p-Value
	Mean (SD)	Mean (SD)		
N	24	23		
Age^a^	32 (8)	35 (12.6)	*t*(37) = −1.119	.270
Gender (female)	0.25	0.35	*χ*^*2*^(1) = 0.537	.464
Education level	4.5 (1.02)	4.57 (0.89)	*t*(45) = −0.232	.817
State detachment	14 (6.90)	9.13 (5.85)	*t*(45) = 2.605	.012
State anxiety^a^	40.04 (10.7)	27.09 (5.8)	*t*(35.6) = 5.213	<.001
PANSS positive	14 (4.3)			
PANSS negative	14 (4.2)			

*Note*. All *p*-values are two sided. Positive and Negative Syndrome Scale (PANSS), seven items positive and seven items negative symptoms subscale.

a Due to inequality of variances the Welch-T test for independent groups was applied.

Before analyses were conducted, it was verified whether individuals were able to correctly identify the emotion in the VR environment after a maximum of two attempts. Trials where individuals failed to correctly identify the emotion of the avatar more than once were excluded from further analyses (excluded trials: SSD group: 7 of 240; comparison group: 8 of 230).^1^

For our control task, which aimed at evaluating differences in task performance between groups for the identity recognition task (Task 1), we conducted one-sided independent samples *t*-tests. The results showed no significant differences in recognition between the SSD group and the comparison group with groups demonstrating a hit rate of 54 % (SSD) and 55 % respectively (comparison). It is important to note that this performance is substantially greater than chance. With 24 avatars in the set and only 10 being targets, a random response pattern would yield a hit rate closer to 10/24, or approximately 42 %. There were neither group differences for hit rates across facial expressions nor for hit rates specifically for angry faces. Further, groups did not differ significantly in their false alarm rates or in measures of sensitivity (d′) or decision bias (c) across all expressions. See Table 2 for details on task performance between groups. Continuity corrections were applied where hit or false alarm rates were either 1 or 0.

**Table 2:** Group differences in task performance.

	SSD	Comparison	Test	p-Value
	Mean (SD)	Mean (SD)		
Task 1 % Hit All Faces	0.54 (0.17)	0.55 (0.19)	*t*(45) = −0.182	.428
Task 1 % False Alarm	0.25 (0.17)	0.2 (0.11)	*t*(45) = 1.325	.096
Task 1 D′	0.9 (0.57)	1.07 (0.66)	*t*(45) = −0.985	.165
Task 1 C-criterion	0.32 (0.47)	0.39 (0.34)	*t*(45) = −0.561	.289
Task 1 % Hit Angry Faces	0.48 (0.38)	0.50 (0.37)	*t*(45) = −0.192	.424
Task 2 % Hit All Faces	0.41 (0.16)	0.47 (0.20)	*t*(45) = −1.156	.127
Task 2 % Hit Angry Faces	0.33 (0.32)	0.48 (0.35)	*t*(45) = −1.479	.073
Task 3 Adjacent Pairs	1.46 (1.47)	1.87 (1.63)	*t*(45) = −0.907	.185

*Note*. All p-values are one sided. SSD: *N* = 24, comparison: *N* = 23.

For our first hypothesis comparing feature binding performance in the identity emotion binding task (Task 2), focusing on the ability to correctly bind emotional expressions to avatars seen in a VR environment, one-sided independent samples *t*-tests did not reveal significant differences between groups across emotions, however, there was a trend-level effect (*t*(45) = −1.48, *p* = .073) for angry faces with the SSD group performing worse (see Table 2).

For the third task, concerning temporal context binding, we could not exclude failed trials as this would have compromised the calculation of the adjacent pair metric, as it requires all trials to produce a valid score. The requirement of complete data also meant that no specific analysis for angry faces alone could be performed. Conducting an independent sample *t*-test on the original measure across emotions, we observed no significant difference between the SSD and comparison group in recalling the order of avatar encounters (see Table 2). Given the relatively low average number of correct adjacent pairs identified in each group (SSD = 1.46, Comparison = 1.87), it can be assumed that the task was challenging for participants in both groups.

To test the second hypothesis, in total four multiple regression models were estimated. First, a model predicting feature binding performance in the identity emotion binding task (Task 2) across emotions, using state detachment as the predictor was estimated (1a). Secondly, the model was run specifically predicting performance for angry faces. Age, gender, education level and state anxiety were included as covariates (2a). For each of these initial models, a second model including performance on the identity recognition task (across emotions and for angry faces respectively) as a covariate was estimated (1b, 2b).

Employing comprehensive modelling across participant groups^2^ while accounting for covariates, we found no effect of state detachment for feature binding performance across emotions, for the model without (1a: *β* = −0.133, *p* = .413, 95 % CI [−0.012, 0.005]) and with identity recognition task performance (Task 1) as a covariate (1b: *β* = −0.143, *p* = .380, 95 % CI [−0.012, 0.005]). However, we found a significant negative association of state detachment and feature binding performance for angry faces (2a: *β* = −0.414, *p* = .011, 95 % CI [−0.036, −0.005]). This effect remained when controlling for the recognition of angry faces in Task 1 (2b: *β* = −0.544, *p* = .002, 95 % CI [−0.044, −0.011]). For visual reference, a scatterplot of the unadjusted relationship between state detachment and angry-face binding accuracy is included in Supplementary Fig. S1, State anxiety did not emerge as a significant predictor of feature binding performance in any of the models.

## 5 Discussion

The present study aimed to investigate the relationship between state detachment and feature binding of avatar characteristics in a virtual reality paradigm using an SSD group and a comparison group from the general population. Based on previous findings, it was hypothesized that individuals with an SSD would display deficits in feature binding compared to the comparison. This hypothesis was not supported by the data. In the recognition task (i.e., controlling for basic memory functioning), no significant differences between groups either across expressions or specifically for angry faces was found. For the identity emotion binding task, which required participants to bind the emotional expression to the identity of the avatars, there was no significant difference in feature binding performance between groups and across facial expressions. While there was a trend for a group difference for angry faces, the difference did not reach statistical significance. Although accuracy was modest, performance exceeded chance levels (e.g., 33 % for SSD vs. 20 % chance in angry-face trials), indicating meaningful engagement. The task was deliberately designed to be challenging, avoiding ceiling effects and enhancing ecological validity. Likewise, there was no significant difference in performance between the two groups in the temporal context binding task in which participants were asked to order avatars (i.e., displaying the correct emotional expression) in accordance to how they appeared in the VR environment.

For the second hypothesis, we investigated whether deficits in feature binding were related to state detachment and whether this effect was specific for angry faces. The model was estimated across groups to increase statistical power, as similar trends in feature binding performance for angry faces and detachment were observed in both the patient and comparison groups, justifying pooled analysis. Whereas state detachment did not appear as a significant predictor of task performance across all emotions, it did emerge as a significant predictor for angry faces specifically. That is, greater state detachment was linked to worse feature binding performance for angry faces in the identity emotion binding task. This relationship remained significant even when controlling for general memory performance (i.e., the recognition ability for angry avatars) and state anxiety.

Although previous research indicated smaller effect sizes for impairment in recognition memory in SSD as opposed to free recall, substantial impairments were nevertheless found (Aleman et al., 1999; Pelletier et al., 2005). In that view, the lack of group differences on the recognition task in the current study were surprising. Further, results of the feature binding tasks are contrasted by previous literature where SSD samples were demonstrated not only to display impairments in contextual binding of information (Diaz-Asper et al., 2008; Rizzo et al., 1996a) but also suggested patients' retrieval to benefit less from contextual cues (Talamini et al., 2010). With respect to temporal context, studies indicate that individuals with an SSD experience it as more challenging to recall the temporal context compared to individuals from the general population (Brébion et al., 2020; Doré et al., 2007; Rizzo et al., 1996b) while our results did not reflect such differences.

The discrepancy between the literature and the lack of group differences observed in this study may be due to several factors. First, the limited number of trials per individual may have hampered our ability to obtain an accurate estimate of an individual's performance. Since the experimental procedure already required around an hour of the patient's time, we did not opt to increase the number of trials even further, particularly because we already anticipated the VR procedure to be straining for participants. Furthermore, our results may have been influenced by the relatively low symptom severity observed in the PANSS scores of our patient group. That is, patients on average scored one standard deviation below positive and negative symptom subscale means identified by Kay and colleagues (Kay et al., 1987). These individuals, stable enough to participate in the complex VR paradigm, may not represent the broader SSD population. Ultimately, it is challenging to determine whether the lack of performance differences between the groups are a result of the design and samples, or due to an absence of real performance differences in feature binding between these two groups.

With regard to the second hypothesis, our results provide tentative evidence for the association between state detachment and feature binding performance for angry faces. While we did not find a group difference in feature binding performance for the identity emotion binding task across emotions, there was a trend level effect for angry faces. Considering this result against the background of significantly higher levels of state detachment in the patient group, it is plausible that the lack of detected group difference could be attributed to a power issue. This is supported by our finding of an inverse relationship between state detachment and feature binding performance for angry faces when combining groups for the analysis, which provided greater statistical power.

These results align with literature describing dissociative symptoms as a means of avoiding or disengaging from adverse stimuli, thus potentially compromising coherent encoding as well as subsequent recall (Ehlers and Clark, 2000; Holmes et al., 2005). While encoding of emotional faces, particularly negative expressions (i.e., angry, fearful), is generally facilitated (Jackson et al., 2009, Jackson et al., 2014; Sessa et al., 2011), this effect appears diminished for angry faces in individuals experiencing state detachment. From an evolutionary perspective, fearful facial expressions may signal looming danger, but they may not represent a direct threat like angry faces and thus may be less likely to evoke state detachment as an avoidance response. This specific effect for adverse stimuli is reflected in our results, as we did not find an association between feature binding performance and state detachment for fearful faces or any other emotional expressions apart from angry faces.

Detaching in response to an adverse event might be an adaptive short-term reaction, allowing individuals to regulate anxiety levels. Typically, increased anxiety and arousal narrow attention to threat-related stimuli (Easterbrook, 1959; Eysenck et al., 2007). However, for some, the experience of state detachment may compromise cognitive processes, such as the ability for coherent binding of features. It is important to note that state detachment was assessed after the paradigm concluded, rather than immediately following exposure to angry faces. This design choice aimed to keep the experience as fluent and immersive as possible. Furthermore, previous research suggests that VR itself can elicit dissociative experiences (Aardema et al., 2010), as such elevated CADSS scores may have been partly influenced by the design. Although state detachment may have been evoked in response to other emotional expressions or the VR setting, we found a specific relationship for angry expressions. A block-wise design with repeated assessments of detachment following exposure to specific emotion types could offer a clearer interpretation. Further, future studies may benefit from including measures of presence or VR-induced dissociation to better isolate these influences. Lastly, it is possible that the causal relationship between impaired feature binding and state detachment is reversed, such that an individual's disjointed memories of an event may bring about an experience of detachment from reality.

### 5.1 Future studies

A promising direction for future experimental research aimed at enhancing our understanding of this mechanism could involve directly manipulating detachment. This can be achieved through methods such as aversive film paradigms (James et al., 2016), which have been effectively utilized in prior studies (Kindt and van den Hout, 2003; Kindt et al., 2005; Giesbrecht et al., 2010) to simulate the emotional effects of adverse events in a more ethical and manageable manner. While these earlier studies focused on temporal sequencing ability for the film clips, it would be particularly intriguing to assess feature binding performance for both neutral and negative elements within these aversive movies. Separating the detachment induction from the comparison of adverse versus neutral stimuli, as in not embedding them into the movie, might constitute an approach with even more experimental control. For instance, using paradigms such as vision deforming glasses (Renard et al., 2018) or a dot staring task (Leonard et al., 1999).

Lastly, it may be beneficial to perform an ecological momentary assessment study to observe naturally occurring (aversive) interactions and their subsequent recall. This approach could also assess perceived or arising detachment in these situations, achieving greater ecological validity than is possible in a VR environment or through aversive movies. By employing these methodological approaches, future studies can provide further insights into the underlying mechanisms and interventions that can effectively address detachment-related memory impairments.

### 5.2 Conclusion

The findings of the present study provide tentative empirical support for the role of detachment in feature binding deficits for angry faces. While we did not find performance difference in feature binding ability between the SSD and comparison group, individuals diagnosed with SSD may nevertheless be more affected due to their comparably high rates of detachment. The objective of subsequent studies will be to gain deeper insights into the potential role that psychological mechanisms such as detachment play in memory dysfunction, in particular with respect to aversive stimuli.

The following is the supplementary data related to this article.

Supplementary Fig. S1 Spearman correlation between state detachment and binding for angry facial expressions. Supplementary Fig. S1

## CRediT authorship contribution statement

**A.J. (Ante) Schlesselmann:** Visualization, Validation, Project administration, Investigation, Formal analysis, Data curation, Conceptualization, Writing – review & editing, Writing – original draft. **G.H.M. (Marieke) Pijnenborg:** Supervision, Software, Resources, Funding acquisition, Conceptualization, Writing – review & editing. **S.A. (Saskia) Nijman:** Software, Resources, Project administration, Funding acquisition, Writing – review & editing, Writing – original draft. **W. (Wim) Veling:** Software, Project administration, Funding acquisition, Writing – review & editing. **R.J.C. (Rafaele) Huntjens:** Validation, Supervision, Project administration, Methodology, Investigation, Funding acquisition, Conceptualization, Writing – review & editing.

## Declaration of competing interest

The development of the VR software was supported by a peer-reviewed €75,000 Knowledge Innovation Mapping grant (628.005.007), awarded by the Netherlands Organization for Scientific Research (NWO). GGZ Drenthe provided €12,173 for four VR headsets and supporting research staff through an internal research grant. Finally, the University of Groningen provided a staff grant of €9,864 for research assistant compensation. None of these institutions have played a role in the design of the study, data collection, analysis, and interpretation of data or in writing the manuscript. All authors declare that they have no competing interests.

## Footnotes

1 Likewise, this logic could be applied to performance in task 1 (identity recognition task) where individuals had to indicate whether they saw the avatar in the VR encoding task. In case an individual reported not having seen an avatar in the VR environment in task 1, it could be anticipated that they were unable to recall its corresponding facial emotional expression in the subsequent task 2 (identity emotion binding task) but instead would have to resort to guessing. However, correcting for this potential confounder by excluding trials would lead to a biassed sample in task 2 consisting of those who were particularly successful in task 1.

2 The model was estimated across groups, since, while the SSD group was expected to display more severe detachment on average than the comparison group, we did not expect detachment and feature binding performance for angry faces to show a differential association between groups. Hence groups were pooled, and the analysis was conducted with increased statistical power across groups. Post-hoc subgroup regression plots displayed similar trends for the association of feature binding performance for angry faces and detachment, although this relationship was stronger in the comparison group.
