# Supplementary figures and images for: Feature binding and detachment in psychosis: A virtual reality study
Source: Schizophr Res Cogn. 2025 Jun 25;42:100376. doi: 10.1016/j.scog.2025.100376 (PMC12246582; doi:10.1016/j.scog.2025.100376)

**Supplements**

Supplement S1


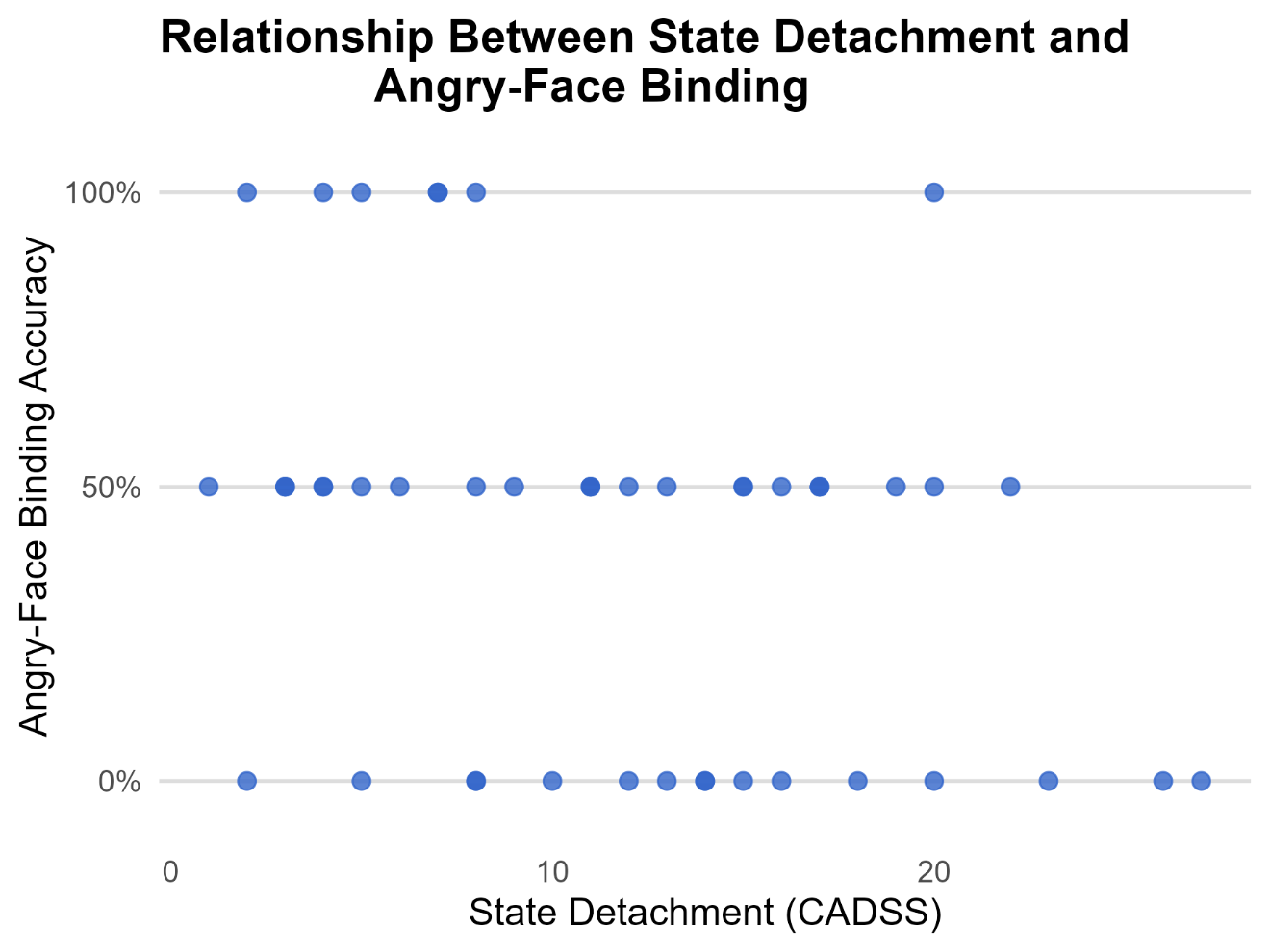


Note: Zero-order spearman correlation *r* = -.326, *p* (two-tailed) = .025.

Supplement: Supplementary Fig. S1 — Spearman correlation between state detachment and binding for angry facial expressions. [file mmc1.docx]
